# Denver Mud

**Published:** 1910-04

**Authors:** 


"DENVER MUD."—When antiphlogistine was put upon the market by the Denver Chemical Co. it was nick-named "Denver Mud," and it came to be extensively known by that name. The Colorado Chemical Co. put upon the market a preparation under that name. The Denver Chemical Co. brought suit against them in the United States Circuit Court at Kansas City and won it; the court enjoined the sale of the stuff advertised as Denver Mud or the use of the name, and ordered that the manufacturers of antiphlogistine "recover from defendants its damages to be assessed as the court may direct," and that the defendant shall pay the costs; further—that defendant shall deliver up all the stuff, labels, circulars, etc., to be destroyed. "So much for Buckingham."

---

ATTENTION is called to Dr. Gregory's splendid lecture reproduced in this issue from the *Houston Chronicle*. It is encouraging to see the big dailies taking an interest in such matters, for the people know nothing of the evils discussed by the doctors, and only through the lay press can they be reached and made to comprehend the necessity for reform. I beg that my readers in every county in Texas will prevail on their local papers to reproduce this forceful address.

